# KC-SMARTR: An R package for detection of statistically significant aberrations in multi-experiment aCGH data

**DOI:** 10.1186/1756-0500-3-298

**Published:** 2010-11-11

**Authors:** Jorma J de Ronde, Christiaan Klijn, Arno Velds, Henne Holstege, Marcel JT Reinders, Jos Jonkers, Lodewyk FA Wessels

**Affiliations:** 1 Department of Bioinformatics and Statistics, The Netherlands Cancer Institute, Plesmanlaan 121, 1066CX Amsterdam, The Netherlands; 2 Department of Molecular Biology, The Netherlands Cancer Institute, Plesmanlaan 121, 1066CX Amsterdam, The Netherlands; 3 Central Microarray Facility, The Netherlands Cancer Institute, Plesmanlaan 121, 1066CX Amsterdam, The Netherlands; 4 Faculty of EEMCS, Delft University Of Technology, 2628 CD Delft, The Netherlands

## Abstract

**Background:**

Most approaches used to find recurrent or differential DNA Copy Number Alterations (CNA) in array Comparative Genomic Hybridization (aCGH) data from groups of tumour samples depend on the discretization of the aCGH data to gain, loss or no-change states. This causes loss of valuable biological information in tumour samples, which are frequently heterogeneous. We have previously developed an algorithm, KC-SMART, that bases its estimate of the magnitude of the CNA at a given genomic location on kernel convolution (Klijn *et al*., 2008). This accounts for the intensity of the probe signal, its local genomic environment and the signal distribution across multiple samples.

**Results:**

Here we extend the approach to allow comparative analyses of two groups of samples and introduce the R implementation of these two approaches. The comparative module allows for a supervised analysis to be performed, to enable the identification of regions that are differentially aberrated between two user-defined classes.

We analyzed data from a series of B- and T-cell lymphomas and were able to retrieve all positive control regions (VDJ regions) in addition to a number of new regions. A t-test employing segmented data, that we implemented, was also able to locate all the positive control regions and a number of new regions but these regions were highly fragmented.

**Conclusions:**

KC-SMARTR offers recurrent CNA and class specific CNA detection, at different genomic scales, in a single package without the need for additional segmentation. It is memory efficient and runs on a wide range of machines. Most importantly, it does not rely on data discretization and therefore maximally exploits the biological information in the aCGH data.

The program is freely available from the Bioconductor website http://www.bioconductor.org/ under the terms of the GNU General Public License.

## Background

### Background and motivation

DNA copy number alterations (CNAs) in tumours are an important mechanism of deregulation of cancer genes. CNAs are a consequence of genomic instability, which is common in human cancers [1]. Various microarray platforms have enabled the genome-wide analysis of CNAs by array based Comparative Genomic Hybridization (aCGH) and many different microarray platforms are currently available for aCGH analysis, including platforms based on bacterial artificial chromosome (BAC) clones, cDNA clones, SNPs and long oligonucleotides. Most of these platforms feature measurement points (probes) at specific positions on the genome with a certain distance between the consecutive probes.

Array CGH data generally consist of the ratios of (log-transformed) intensities of fluorescently labeled DNA from case (disease) versus normal diploid (2 n) control samples that are measured by the probes on the array. Although single cell aCGH analysis is possible [2] most aCGH analyses are performed on samples derived from tissue which contains sub-populations of different cells. This implies that an aCGH measurement will measure the average of CNAs of different sub-populations within the sample. Therefore, discretization of the data may lead to the loss of valuable biological information. KC-SMARTR does not discretize the data and makes use of the continuous signal to preserve all the information contained in the data. The software package allows unsupervised analysis to identify recurrent aberrations across samples as well as supervised analysis to identify regions that are differentially aberrated between user defined classes of samples. These analyses are two of the most commonly performed on aCGH data and KC-SMARTR combines them in one, easy to use and flexible program.

## Implementation

### Unsupervised KC-SMART

To identify regions which are significantly aberrated the KC-SMART method [3] takes into account 1) the non-discretized signal intensity of a probe; 2) the strength of neighboring probes and 3) the strength of the probe across multiple samples. These steps are performed separately for the gains and losses. First, the probe intensities are summed across all samples. Next, kernel convolution is performed across the genome, along with locally weighted regression to account for unequally distributed probes. This results in a kernel smoothed estimate of probe intensities, the 'KC score'. The size of the kernel has consequences for the type of aberration that will be detected by the algorithm (see next section). Finally, the significance threshold is determined using a permutation based approach and significant aberrations are defined as the set of probes for which the KC score exceeds this threshold. The set of genomic scales ranging from the smallest to the largest kernel width is defined as the 'scale space'. The KC-SMART analysis is repeated for a selection of kernel widths from the scale space to reveal the aberrations that are significant at different genomic scales.

**Figure 1:**
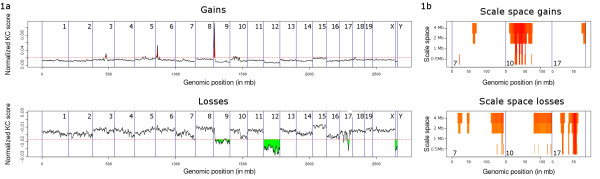
**Genome-wide plot of the KC scores of a Nimblegen mouse data set (Klijn C, et al. Unpublished data 2008) using a kernel width of 1 Mb, the red dotted line indicates the significance threshold determined using an alpha cut-off of 0.05**. A large gain on chromosome 9 and the loss of chromosome 12 clearly stand out, reflecting both the strength and the frequency of the respective gain and loss. **b) **Scale space plot, showing the significant regions on chromosomes 7, 10 and 17 for four different kernel widths where the color indicates the level of significance (ranging from red to yellow where red indicates highly significant aberrations and yellow less significant aberrations).

The R implementation that we introduce here permits calculation of significantly recurrent gains and losses from aCGH data and features a graphical overview of these gains and losses (Figure 1a). In addition, the probes residing in these regions can be retrieved in a tabular format. Significantly recurrent aberrations are identified across the scale space, and the results of this analysis are combined in one graphical overview (Figure 1b). Varying the kernel width allows analysis on different biologically relevant scales: a large kernel width will show gains and losses over large (sub-chromosomal) regions while a small kernel width will allow the detection of smaller gains and losses (kilobase or megabase regions). Obviously, the minimal size of gains and losses that can be detected also depends on the resolution of the (aCGH) platform used to measure the signal. The kernel width, the resolution (i.e. the number of points sampled from the convoluted kernels) and the significance threshold level are all user selectable.

### Supervised KC-SMART

In addition to the single class analysis aimed at finding recurrent CNAs, KC-SMARTR also features a new, supervised approach to perform a comparative analysis, i.e. it allows the direct comparison of two groups of samples. This allows the detection of regions representing significant, differential copy number changes between groups, i.e. class-specific CNAs. In contrast to the unsupervised KC-SMART approach which performs a kernel convolution on the summed ratios of the tumor set, the comparative approach performs a kernel convolution on each *individual *tumor profile, resulting in a KC score for each sampling point for each sample. Then two alternative analysis routes can be followed. In the first approach, we compute, for each genomic position (sampling point), *i*, the signal-to-noise ratio:

(1) $$SNR(i)=\frac{\mu_{KC^{1}}(i)-\mu_{KC^{2}}(i)}{\sigma_{KC^{1,2}}(i)+f}$$

where μ_KC_^1^(*i*) and *μ*_KC_^2^(*i*) are the averages of the KC scores at position *i *over all samples in Groups 1 and 2, respectively; σ_KC_^1,2^(*i*) is the pooled variance over all samples of the KC scores at position *i*, and *f *is a regularization factor equal to the 95^th ^percentile of the pooled class standard deviation across all genomic positions. This factor prevents small variances from dominating the SNR statistic. To identify significantly differential CNAs, a class label based permutation scheme is employed to determine the SNR threshold that satisfies the user-specified false discovery rate. In the second approach, the smoothed tumor profiles are employed as input to the SAM package [4], to identify differentially aberrated loci at a given FDR.

## Results

**Figure 2:**
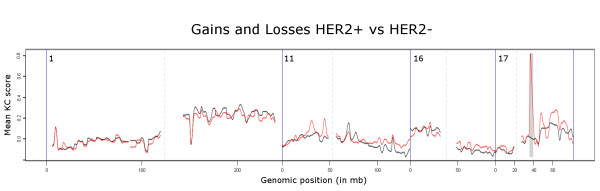
**shows the visual output of the comparative analysis of KC-SMARTR, run on the breast cancer aCGH dataset from Chin **[5]**comparing the HER2-positive group (red) to the HER2-negative group (black)**. A kernel width of 1 Mb and an FDR cut-off of 0.01 were used. The chromosome 17q amplicon, characteristic for HER2-positive tumors, stands out clearly (the significant region as determined by the SNR algorithm is indicated as a grey shaded area).

**Figure 3:**
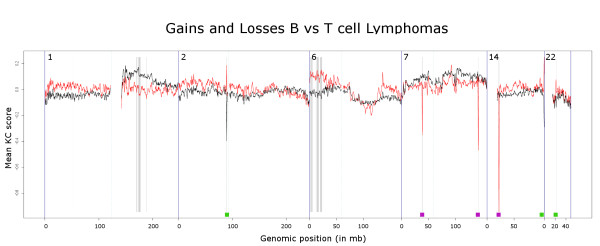
**shows the comparative KC-SMARTR graphical output, run on the B- and T-cell lymphoma dataset**. The black line represents the B-cell lymphomas and the red line the T-cell lymphomas. Using a kernel width of 200 kb and an FDR of 5%, the VDJ regions can clearly be distinguished as significantly lost in the B-cells on chromosome 2, 14 and 22 and as significantly lost in the T-cells on chromosomes 7 and 14. The green bars indicate the approximate positions of the immunoglobulin variable regions, the purple bars indicate T-cell receptor variable regions. Also see Table 1 for a list of identified regions over the entire genome.

Figure 2 shows an example of the visual output from the comparative KC-SMARTR analysis of a publicly available breast cancer aCGH dataset [5] in which the 17q amplicon (containing the *HER2 *gene) is clearly identified as a significant differential CNA in the HER2-positive breast cancer group. In a recent cross species comparison study [6] our algorithm was used successfully to compare mouse to human aCGH data, showing the wide range of datasets our method can be applied to. For a more in depth analysis and comparison of KC-SMARTR to other methods we made use of a publicly available aCGH dataset [7] consisting of copy number profiles of cell lines derived from B- and T-cell lymphoma's. B- and T-cells are subject to somatic VDJ recombination at the immunoglobulin and T-cell receptor (TCR) loci, respectively. B- and T-cell lymphomas will therefore have clonal VDJ recombinations characterized by regional copy number losses that are specific to the cell type and provide a positive control in our analysis. In order to evaluate the KC-SMARTR method and to exploit these intrinsic positive controls, we divided the data into two groups: a group consisting of B-cell lymphomas and a group of T-cell lymphomas. Given the fact that VDJ recombination takes place we would expect the B-cell lymphomas to have lost these variable regions on chromosomes 2, 14 and 22, compared to the T-cell lymphomas. Conversely, the T-cell lymphomas would be expected to show lost regions on chromosomes 7 and 14. We expect the rearrangements at the T and B-cell loci to be small, so we chose to perform the analysis using a small (200 kb) kernel width. We were able to recover exactly those regions that are subject to VDJ recombination as significantly aberrated regions (See figure 3). In addition to these regions we also found significantly aberrated regions on chromosomes 1 and 6 (See Table 1).

**Table 1:** This table shows the regions that were identified by KC-SMARTR as being significantly aberrated in the B- and T-cell lymphoma dataset.

Chromosome	Region (in kb)	Known VDJ loci in region
***1***	51300 - 51300	-
***1***	168900 - 170100	-
***1***	171600 - 171900	-
***1***	172800 - 176700	-
***1***	187500 - 188100	-
***2***	88800 - 89400	Ig* Kappa light chain
***6***	1800 - 4200	-
***6***	5400 - 6300	-
***6***	11100 - 11100	-
***6***	13200 - 17100	-
***6***	19800 - 23100	-
***6***	24300 - 24300	-
***7***	38100 - 38700	T-cell receptor Gamma
***7***	141900 - 142200	T-cell receptor Beta
***14***	21300 - 22200	T-cell receptor Alpha
***14***	105300 - 105900	Ig heavy chain
***22***	21300 - 21600	Ig Lambda light chain

*Ig - Immunoglobulin

All positive control regions (i.e. the regions that are known to be involved in VDJ recombination) were identified. Additionally, five regions on chromosome 1 and six regions on chromosome 6 were found.

**Figure 4:**
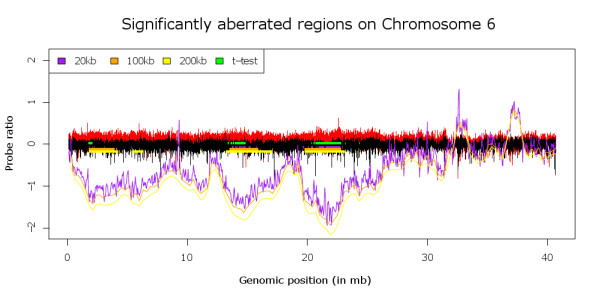
**shows the probe ratios for the T- and B-cell lymphoma data (in red and black respectively)**. The KC-SMARTR profiles (lines) and significant regions (blocks) using different kernel widths are shown in different colors (see legend for details). In green the regions that are reported as significant by the t-test on segmented data are shown. The larger kernel widths (100 kb and 200 kb) allow the detection of larger regions whereas the smaller kernel width (20 kb) allows the detection of smaller regions. In this way the data can be analyzed on different scales. In contrast, the t-test only reports regions on a single scale.

To the best of our knowledge, there is no other publicly available method capable of performing a comparative aCGH analysis. We therefore decided to compare our method against a t-test on segmented data. In this approach we segment our data using the DNAcopy package [8] and perform a t-test between the two defined groups (i.e. B-cell lymphomas versus T-cell lymphomas) using the segment values at each probe location, which returns a t-statistic for each probe. To control the false discovery rate (FDR) we employ the SAM package to identify significant probes. The significant probes are then combined into significant regions which can be compared to the regions as identified by KC-SMARTR. Using an FDR setting of 5% the resulting regions contained all the VDJ control regions and several other regions, both overlapping and non-overlapping with the regions identified by KC-SMARTR [Additional file 1: Supplemental Table S1]. The amount of scattering (i.e. many small regions within a larger region are reported) may depend on the settings of the segmentation algorithm and the false discovery rate employed for the t-test. To avoid having to optimize these settings for every approach, and in the process most likely overfitting the data and thus biasing the approaches towards a desired result, we employed the DNAcopy default settings and used an FDR setting of 5% for the t-test. At these default settings many of the reported regions are highly scattered. This is in contrast to the results from the KC-SMARTR analysis which features smoothing of the data and incorporates data from neighboring probes (see Figure 4), a difference that is also reflected in a higher median sensitivity (91% versus 69%, specificity 15% vs 31% [Additional file 1: Supplemental Table S2). Herein lies the strength of the KC-SMARTR approach, that the user can select the appropriate kernel width to identify aberrated regions of relevant size. The kernel width can be chosen such that noisy data will be smoothed but small aberrations are reliably detected. Conversely, larger kernel widths can enable the detection of broader, lower amplitude gains and losses. This is an important advantage over the t-test on segmented data that in our example returns a very fragmented aberrated region that may not correspond to the actual copy number within those regions. To assess whether the regions identified by KC-SMARTR that are located outside of known VDJ-regions are indeed important in tumorigenesis, further functional experiments would be needed.

## Discussion

To the best of our knowledge no other software package exists that allows for a supervised aCGH analysis and as such we believe our method delivers an important contribution to this field. Also, given the fact that the method does not make use of discretized data, for recurrent gain and loss analysis the software gives the user the flexibility to look for aberrations across different genomic scales. Given the ever increasing data set sizes it is also important to note that our algorithm scales linearly with the number of probes and number of samples. To give an indication, on our Opteron 2.7 GHz the analysis of a fairly large Affymetrix SNP 6 (1.78 Million probes) dataset consisting of 61 samples a comparative analysis took about five and a half hours.

In the future we would like to implement a parallelized algorithm to make use of additional cpu cores that are frequently available in current machines. This would speed up the process a lot since most calculations can be performed in parallel.

## Conclusions

KC-SMARTR is a flexible, fast and user-friendly aCGH tool to determine significantly recurrent CNAs as well as regions showing significantly differential aberrations between two groups of samples. On a set of B- and T-cell lymphomas we were able to locate all positive control regions (VDJ recombination sites) and a number of new regions as significantly aberrated. A t-test run on segmented data was also able to find the positive control regions but resulted in highly fragmented regions. In contrast, KC-SMARTR allows the user to set the kernel width and thereby control the size of the aberrations that are detected. It features output in both visual and tabular format, including a scale space analysis, which allows a visual overview of the aberrations at different scales. KC-SMARTR offers recurrent CNA and class specific CNA detection, at different genomic scales, in a single package without the need for additional segmentation. It is memory efficient and runs on a wide range of machines. Most importantly, it does not rely on data discretization and therefore maximally exploits the biological information in the aCGH data.

## Availability and requirements

**Project name**: KC-SMART

**Project home page**: http://bioconductor.org/packages/2.5/bioc/html/KCsmart.html

**Operating system(s)**: Platform independent

**Programming language**: R

**License**: GNU General Public License

**Installation note**: To always get the most up-to-date version of KC-SMARTR, follow the procedure below. Update to the latest R and Bioconductor version and type the following at the R prompt: source ("http://bioconductor.org/biocLite.R") biocLite("KCsmart")

## Competing interests

The authors declare that they have no competing interests.

## Authors' contributions

JdR participated in the design of the study, wrote code for the software package, was involved in the analyses and wrote the manuscript, CK and AV participated in the design of the study, wrote code for the software package and was involved in the analyses, HH participated in the design of the study, MR, JJ and LW participated in the design of the study and conceived of the study. All authors have read and approved the final manuscript.

## Supplementary Material

Additional file 1 **Supplemental Data**. Contains Supplemental Table S1 and Supplemental Table S2. Click here for file
