# Supplementary material for: KC-SMARTR: An R package for detection of statistically significant aberrations in multi-experiment aCGH data
Source: BMC Res Notes. 2010 Nov 11;3:298. doi: 10.1186/1756-0500-3-298 (PMC2995794; doi:10.1186/1756-0500-3-298)
Supplement: Additional file 1 — Supplemental Data. Contains Supplemental Table S1 and Supplemental Table S2. [file 1756-0500-3-298-S1.DOCX]

# supplemental data

Supplemental Table S1

| **Chromosome** | **Region (in kb)** | **Known VDJ loci in region** |
| --- | --- | --- |
| ***1*** | 51156 - 51229 | *Non-overlapping with KC-SMARTR* |
| ***1*** | 169933 - 169936 | - |
| ***1*** | 173069 - 173463 | *Non-overlapping with KC-SMARTR* |
| ***2*** | 88914 - 89282 | Immunoglobulin (Ig) Kappa light chain |
| ***6*** | 1754 - 1919 | - |
| ***6*** | 1923 - 2043 | - |
| ***6*** | 13394 - 13397 | - |
| ***6*** | 13400 - 13413 | - |
| ***6*** | 13599 - 13743 | - |
| ***6*** | 13814 - 13896 | - |
| ***6*** | 13972 - 14053 | - |
| ***6*** | 14099 - 14420 | - |
| ***6*** | 14423 - 14824 | - |
| ***6*** | 20345 - 20355 | - |
| ***6*** | 20525 - 20593 | - |
| ***6*** | 20707 - 22769 | - |
| ***6*** | 23172 - 23172 | *Non-overlapping with KC-SMARTR* |
| ***6*** | 24346 - 24357 | *Non-overlapping with KC-SMARTR* |
| ***7*** | 38261 - 38365 | T-cell receptor Gamma |
| ***7*** | 70059 - 70061 | *Non-overlapping with KC-SMARTR* |
| ***7*** | 141821 - 142204 | T-cell receptor Beta |
| ***8*** | 128836 - 128847 | *Non-overlapping with KC-SMARTR* |
| ***12*** | 9543 - 9607 | *Non-overlapping with KC-SMARTR* |
| ***14*** | 21439 - 22047 | T-cell receptor Alpha |
| ***14*** | 105290 - 105601 | Ig heavy chain |
| ***14*** | 105641 - 105854 | Ig heavy chain |
| ***14*** | 105867 - 105867 | Ig heavy chain |
| ***14*** | 105887 - 105943 | Ig heavy chain |
| ***14*** | 105994 - 106005 | *Non-overlapping with KC-SMARTR* |
| ***14*** | 106015 - 106105 | *Non-overlapping with KC-SMARTR* |
| ***14*** | 106204 - 106239 | *Non-overlapping with KC-SMARTR* |
| ***14*** | 106246 - 106251 | *Non-overlapping with KC-SMARTR* |
| ***14*** | 106269 - 106287 | *Non-overlapping with KC-SMARTR* |
| ***14*** | 106356 - 106356 | *Non-overlapping with KC-SMARTR* |
| ***19*** | 5461 - 5462 | *Non-overlapping with KC-SMARTR* |
| 22 | 21429 - 21552 | Ig Lambda light chain |

Supplemental Table S1. This table shows the regions that were identified by applying a SAM analysis on DNACopy segmented data using an FDR cutoff of 5%. Just like the KC-SMARTR analysis all positive control regions (i.e. the regions that are known to be involved in VDJ recombination) were identified. Additionally, this approach yielded a number of regions that are non-overlapping with the regions identified by KC-SMARTR (200kb kernel width). The majority of the non-overlapping regions are located close to regions that are reported by KC-SMARTR. Furthermore, the significant regions are much more fragmented than the significant regions resulting from the KC-SMARTR analysis.

Supplemental Table S2

|  | **KC-SMARTR** | **Segmented t-test** |
| --- | --- | --- |
| ***Median sensitivity*** | 91% | 69% |
| ***Median specificity*** | 15% | 31% |

Supplemental Table S2. This table shows the median sensitivity and specificity in reporting known VDJ regions (the six regions shown in table 1). These regions are defined by taking the maximum spanning regions containing VDJ genes as reported by the UCSC genome browser, build 36. The sensitivity is then defined as the percentage of base pairs of these maximum spanning regions covered by the KC-SMARTR or segmented t-test significant regions respectively. The specificity is defined as the percentage of base pairs from the KC-SMARTR and segmented t-test regions that fall inside these defined regions (only regions close to or (partly) covering the VDJ regions are included, i.e. chromosomes 1 and 6 are not taken into account in this calculation).
